# Novel pharmacotherapy: NNI-362, an allosteric p70S6 kinase stimulator, reverses cognitive and neural regenerative deficits in models of aging and disease

**DOI:** 10.1186/s13287-020-02126-3

**Published:** 2021-01-13

**Authors:** Nathalie Sumien, Matthew S. Wells, Akram Sidhu, Jessica M. Wong, Michael J. Forster, Qiao-Xi Zheng, Judith A. Kelleher-Andersson

**Affiliations:** 1 Department of Pharmacology & Neuroscience, University of North Texas Health Science Center, 3500 Camp Bowie Blvd., Fort Worth, TX USA; 2 Neuronascent, Inc., 15601 Crabbs Branch Way, Rockville, MD 20855 USA; 3 Novavax, 21 Firstfield Rd., Gaithersburg, MD 20878 USA

**Keywords:** Alzheimer’s disease, mTOR/p70S6 kinase axis, Allosteric modulator, Human neural progenitors, Hippocampal neuron regeneration, BrdU^+^ neuron survival

## Abstract

Aging is known to slow the neurogenic capacity of the hippocampus, one of only two mammalian adult neurogenic niches. The reduction of adult-born neurons with age may initiate cognitive decline progression which is exacerbated in chronic neurodegenerative disorders, e.g., Alzheimer’s disease (AD). With physiologic neurogenesis diminished, but still viable in aging, non-invasive therapeutic modulation of this neuron regeneration process remains possible. The discovery of truly novel neuron regenerative therapies could be identified through phenotypic screening of small molecules that promote adult-born neurons from human neural progenitor cells (hNPCs). By identifying neuron-generating therapeutics and potentially novel mechanism of actions, therapeutic benefit could be confirmed through in vivo proof-of-concept studies. The key aging and longevity mTOR/p70S6 kinase axis, a commonly targeted pathway, is substrate for potential selective kinase modulators to promote new hippocampal neurons from NPCs. The highly regulated downstream substrate of mTOR, p70S6 kinase, directly controls pleiotropic cellular activities, including translation and cell growth. Stimulating this kinase, selectively in an adult neurogenic niche, should promote NPC proliferation, and cell growth and survival in the hippocampus. Studies of kinase profiling and immunocytochemistry of human progenitor neurogenesis suggest that the novel small molecule NNI-362 stimulates p70S6 kinase phosphorylation, which, in turn, promotes proliferation and differentiation of NPCs to neurons. NNI-362 promoted the associative reversal of age- and disease-related cognitive deficits in aged mice and Down syndrome-modeled mice. This oral, allosteric modulator may ultimately be beneficial for age-related neurodegenerative disorders involving hippocampal-dependent cognitive impairment, specifically AD, by promoting endogenous hippocampal regeneration.

**Supplementary Information:**

The online version contains supplementary material available at 10.1186/s13287-020-02126-3.

## Introduction

Neurogenesis in adult humans is an accepted phenomenon following demonstration of BrdU^+^ neurons in the hippocampal dentate gyrus (DG) [1]. However, neurogenesis, in aged individuals and chronic neurodegenerative conditions, remains disputed [2]. An article suggested a lack of measurable human neurogenesis with age [3], while data support continuous neurogenesis throughout aging [4, 5] through neurogenic slowing where differentiating neural progenitors may have longer latency phase before becoming functional neurons [6]. Neural stem cell markers appeared age-independent, while proliferative and maturation factors diminished with age, but independently of each other, providing multiple neural targets for intervention [7]. In AD postmortem DG, neurogenesis was reduced and co-neuron maturation factors dropped-off in relation to later Braak stages [8]. The reduced maturation of differentiated early neurons “in waiting” may be causative of age-related dementias [6] and suggests the later stages of neuron maturation are age-critical targets. Antidepressants promoting neuronal progenitor proliferation [9] have minimal benefit on depression in aged patients [10, 11], suggesting that enhanced progenitor proliferation is insufficient to promote functioning new neurons. With the continued presence of neural progenitors in aged subgranular zone, this niche remains a putative pharmacotherapeutic target for promoting adult-born neurons.

The standard therapeutic-tested Down syndrome (DS) model, Ts65Dn mice, exhibits many phenotypes of DS and AD [12]. Though Ts65Dn has no direct APP gene-dosage-linked pathology, other trisomy-related phenotypes provide abundant rationale for testing therapeutics to address memory and neurogenic dysfunction [13]. Reversing the neurogenic deficit in young Ts65Dn mice using fluoxetine provided a memory benefit [14, 15], though the effects’ durability in adults remains questionable [16].

New neuron formation in neurodegenerative disorders suggests potential for neuro-compensation as observed in postmortem AD tissue [17], Huntington’s patients [18], and stroke in non-human primates [19]. Whether therapies targeting this *compensatory neurogenesis* are effective in age-related neurodegenerative disorders (ARND) remains undetermined. One non-pharmacotherapy promoting neurogenesis and cognition in aging is aerobic exercise. Human trials demonstrated a 6- or 12-month aerobic fitness program improved cognition, increased hippocampal volume [20, 21], and promoted adult-born neurons to compensate for the loss in the anterior hippocampus [20]. Apparently, the brain retains an innate ability for neuron regeneration even in ARND. Since aging is the greatest risk factor for AD [22], any purported disease-modifying AD pharmacotherapy must be effective in compensating for the slowing of adult-born neuron formation and the associated cognitive decline in aging and AD.

The exact [non-therapeutic] trigger that initiates compensatory neurogenesis in ARND is unknown, though growth factors and downstream signaling pathways appear likely candidates. Growth factor mimetics have been tested clinically, exhibiting side effect profiles outweighing any direct gains. However, working downstream at the mTORC1/p70S6 kinase axis would provide a more precise control of protein synthesis and neuron regeneration. Moreover, p70S6 kinase stimulation requires phosphorylation at eight sites, including four auto-inhibitory pseudo-substrate sites [23]. Inhibitors of kinases upstream of p70S6 kinase show benefit as age-related disorder therapeutics, though often associated with toxic side-effects [24]. Without clear/safe targets for promoting adult-born functional neurons, the use of a phenotypic screening platform using hNPCs, and small molecule libraries is a favorable option. The ability to measure neural progenitor proliferation and differentiation to mature neurons in culture has been suggested as necessary to identify novel therapies [25]. Our aim was to discover orally administrated, novel kinase modulators that penetrate the BBB, and promote the formation of functional adult-born neurons from endogenous neural progenitor cells.

## Results

**Fig. 1:**
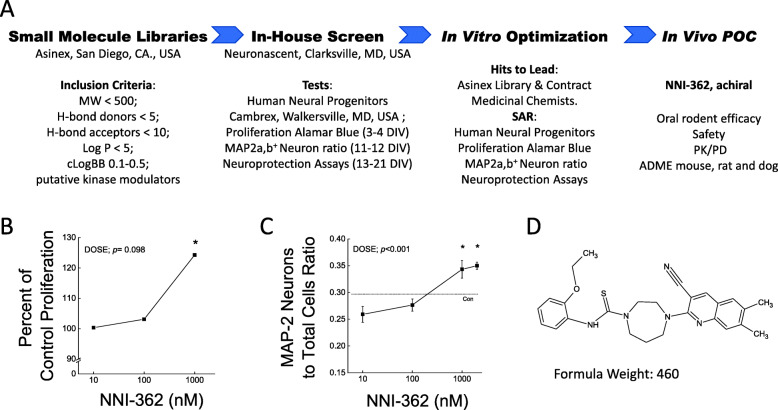
Discovery of neuron restorative small molecule therapy, NNI-362. Phenotypic screen to measure both proliferative and neuron maturity capacity from commercial novel libraries (**a**); proliferation using Alamar Blue, *n* = 5 (**b**). Ratio of mature neuron (MAP2a,b^+^) to total cell numbers (Hoechst dye) from human neural progenitors at DIV12, *n* = 6–12. Structure and formula weight of NNI-362 (**d**). Oxidative cellular metabolism directly related to cell numbers was measured by fluorescence increase in Alamar Blue using emission filter 530/580 nm. Data were analyzed using Kruskal-Wallis with Group as factor. *p < 0.05 vs. controls

Neuronascent’s phenotypic screening platform using hNPCs (Fig. 1a) allowed the discovery of novel neurogenic agents that are also neuroprotective. Due to in silico physicochemical characteristics provided by the commercial library vendor, the number of screened molecules can be narrowed to only BBB-permeable and drug-like compounds. This is critical for small company drug discovery programs in order to quickly progress from the in vitro screening into in vivo efficacy testing. This platform description was expanded from that described by Kelleher-Andersson [25] and revealed a lead candidate: NNI-362 (Fig. 1d). NNI-362 promoted proliferation at 1000 nM versus vehicle (*p* < 0.05) at DIV3 (Fig. 1b) and increased the ratio of mature neurons (MAP2a,b^+^) to total cells at DIV12/13 at ≥ 1000 nM versus vehicle (all *p* < 0.05) (Fig. 1c).

**Fig. 2:**
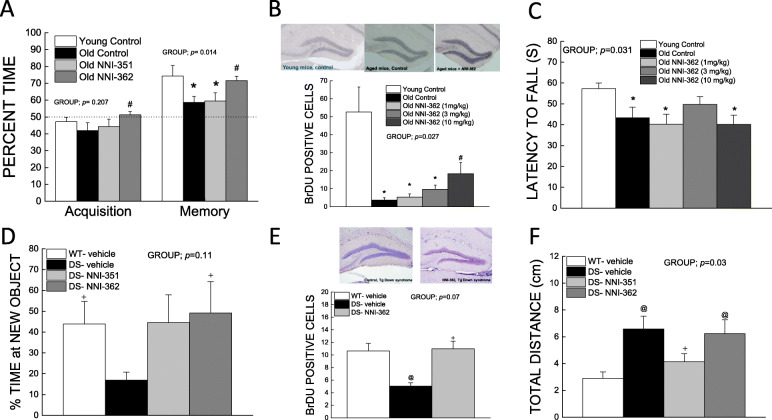
NNI-362 improves memory and increases adult-born neural cells in an aging mouse model (**a**–**c**) and a Down syndrome mouse model (**d**–**f**). Percent time spent with a novel object during acquisition and memory task, *n* = 8–11 (**a**). Number of BrdU-positive cells in the dentate gyrus of the hippocampus after 5–6 weeks oral treatment, *n* = 3 (**b**). Latency to fall from bridge test, *n* = 8–11 (**c**). Percent time spent with a novel object during memory task, *n* = 5–9 (**d**). Number of BrdU-positive cells in the dentate gyrus of the hippocampus after 4 weeks oral treatment, *n* = 2–4 (**e**). Distance traveled during an open field test, *n* = 9–14. BrdU staining and DS behavioral outcomes were analyzed using Kruskal-Wallis with group as factor. Behavioral measurements for the aging study were subjected to one-way ANOVA with group as factor. *p < 0.05 vs. young control; ^#^*p* < 0.05 vs. old control; ^@^*p* < 0.05 vs. WT-vehicle; ^+^*p* < 0.05 vs. DS-vehicle

Young mice spent more time with the novel object than old mice (*p* < 0.05) and old NNI-362-treated mice exhibited a similar pattern to the young mice (*p* < 0.05 vs. old controls) (Fig. 2a). Old mice had lower new BrdU^+^ cells (*p* < 0.05), while the old 10 mg/kg NNI-362 dose had higher BrdU^+^ cells migrating/surviving than the old controls (*p* < 0.05) and were not significantly different from the young (*p* = 0.08) (Fig. 2b). This effect was associated with an improvement in memory function only at the 10 mg/kg dose (data not shown) similar to Fig. 2a. NNI-362 did not reverse age-related motor dysfunction (Fig. 2c). Similarly, in Ts65Dn mice, NNI-362 treatment was associated with reversal of DS-modeled memory impairment (*p* < 0.05) (Fig. 2d) and decreased number of mature BrdU^+^ cells surviving in the DG (*p* < 0.05) (Fig. 2e). NNI-362 did not reduce the hyperactivity observed in Ts65Dn mice (Fig. 2f). NNI-351 was also discovered through the phenotypic screen (Fig. 1a); however, it lacked the neuroprotection capacity in vitro and did not improve memory during aging or consistently in the DS model (Fig. 2), yet did reduce hyperactivity (Fig. 2f).

**Fig. 3:**
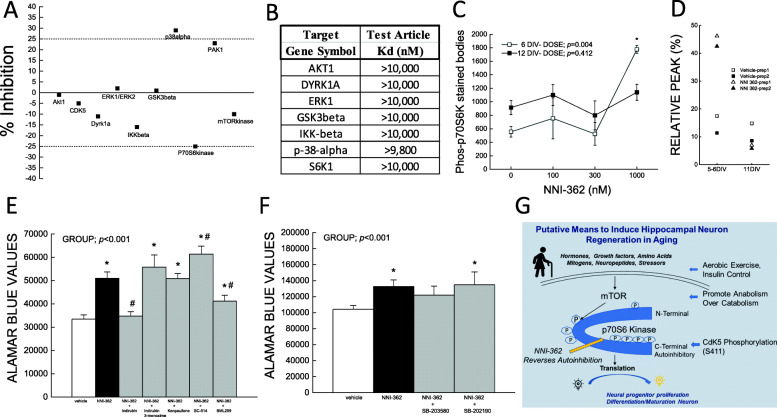
NNI-362 putative mechanism via allosteric stimulation. Significant kinases that either are inhibited or stimulated by NNI-362 in CEREP panel of 151 kinases, significant with ± 25% inhibition (**a**). Measure of active site competition serial dilution to 10× (1000 nM) the effective concentration of NNI-362 (DiscoverX, KinomeScan) (**b**). Phosphorylation of p70S6 kinase in cultures prepared from 6 and 12DIV human neural progenitors treated with 0, 100, 300, and 1000 nM NNI-362 with each media change, *n* = 3 (**c**). Effect of NNI-362 on p70S6 kinase in two separate preparations of human neuronal progenitor cells at 5–6 and 11DIV (**d**). Phosphorylated kinases not consistently modulated by NNI-362: Akt1/2/3&pan, CREB, ERK1/2, GSK3beta, HSP 27, JNK1/2&pan, MKK3, MKK6, MSK2, p38alpha/beta/delta/gamma, p53, RSK1/2, and TOR. Effects of CDK5 inhibitors (indirubin and BML529), GSK3beta inhibitors (indirubin-3-monoxime and kenpaullone), and IKK2 inhibitor (SC-514) (**e**) on NNI-362-associated proliferation. Effects of p38 MAPK inhibitors on NNI-362-associated proliferation (**f**). Putative mechanism describing neuronal regeneration during aging and potential mechanism of action for NNI-362 (**g**). In vitro studies were analyzed using Kruskal-Wallis with group as factor. *p < 0.05 vs. vehicle, ^#^*p* < 0.05 vs. NNI-362

NNI-362 only significantly stimulated the p70S6 kinase from the 151 kinase panel (Fig. 3a). No competition at the active site of p70S6 nor any of the other pathway-related kinases were observed, providing evidence for allosteric targeting by NNI-362 (Fig. 3b). Only at the neuron regenerative concentration (≥ 1000 nM) and only during the early dividing and beginning differentiation phase was p70S6 kinase selectively phosphorylated (6DIV) significantly (*p* < 0.05), while being absent in fully differentiated neurons at 12DIV (Fig. 3c, d). The switching on of translation and increased neurogenesis is possible through numerous targeting points of the mTOR/p70S6 kinase axis [23], but selectivity to *neural* cells may be only possible by means of predominantly neuronal CDK5 kinase phosphorylation at p70S6 kinase auto-inhibitory site [26]. Results confirm only the addition of CDK5 inhibitors, indirubin and BML259, ameliorated NNI-362-induced proliferation, while the addition of other inhibitors, including p38 kinase inhibitor, was ineffective (Fig. 3e, f). These results suggest that NNI-362 acts at the auto-inhibitory pseudo-substrate site, Ser411, where CDK5 phosphorylates p70S6 kinase (Fig. 3g) during the mitogenic translational stage, not the terminally differentiated neuron stage.

## Discussion

The main findings from this study were that NNI-362 promoted proliferation and survival of adult-born neural cells associated with reversal of cognitive deficits, without toxic or off-target effects up to 6 weeks of administration, and acts allosterically and downstream of mTOR.

The screening platform allowed for the discovery of small molecule therapies that promote proliferation and increase new mature neuron numbers in culture, through unique kinase modulating pathway(s). This phenotype is obligatory for the compound to succeed as a human therapy for age-related degenerative disorders. A therapeutic candidate that is only neural progenitor-proliferative appears unable to reverse age-related cognitive impairment [11]. Fluoxetine had neurogenic and behavioral benefit in young TS65Dn mice [14, 15], but not in adult mice [16]. Noteworthy, NNI-351, a compound with only a proliferative phenotype (Ki67^+^ cells greater than DS-vehicle, data not shown), reduced hyperactivity, but was ineffective in reversing memory deficits in aging and DS models, suggesting that maturation and survival of adult-born neural cells are necessary for cognitive improvements in aging and neurodegenerative disease. Indeed, brain aging may be more complex especially in the adult hippocampal neurogenic niche, where “stem cell exhaustion” [27] may be less critical than promotion of maturation of dividing neural progenitors/early neurons to fully mature, functioning neurons. Boldrini et al. [4] suggest the human neural progenitor hippocampal pool remains available for neurogenesis into our 80s. This need for promotion of maturation of the adult-born neurons appeared more critical in AD than aging alone [8]. Further studies of differentiation and synaptic integration will be needed to conclusively resolve that NNI-362 has an effect on neurogenesis beyond proliferation, migration, and adult-born cell survival.

Identifying the mechanism of action of NNI-362 by which neural cells proliferate, migrate, survive, and improve cognitive function could lead to novel target identification for disease-modifying neuron regeneration. While a selective p70S6 kinase inhibitor is known [28], our data indicate that NNI-362 stimulates p70S6 kinase, suggesting activity at a critical auto-inhibitory allosteric site: Ser411 (conserved across many species [29]), a substrate for CDK5/activator phosphorylation, suggesting selectivity to neural tissue [30]. Inhibition of CDK5 ameliorated NNI-362-induced proliferation accounting for NNI-362’s neuron selectivity, while other upstream kinases or their inhibitors played no definitive role in induced neuron generation.

## Conclusion

Elucidating the CDK5/activator substrate Ser411 site on p70S6 kinase [26] that is selectively stimulated in neural cells by NNI-362 provides greater insights into how this clinical-stage therapeutic works in aging brain and neurodegenerative disorders. Further studies will look at selective neuron regeneration markers and reversal of cognitive deficits in aging and neurodegenerative models and the level of allosteric site stimulation on p70S6 kinase by Western and MAP kinase profiling in vivo. Though mTOR *inhibition* has been suggested to promote longevity, our data support a downstream target to improving brain “healthspan”: restorative capacity via neural-selective allosteric stimulation of p70S6 kinase.

## Supplementary Information

**Additional file 1.**

## Data Availability

The datasets used and/or analyzed during the current study are available from the corresponding author on reasonable request.
