# Supplementary material for: Novel pharmacotherapy: NNI-362, an allosteric p70S6 kinase stimulator, reverses cognitive and neural regenerative deficits in models of aging and disease
Source: Stem Cell Res Ther. 2021 Jan 13;12:59. doi: 10.1186/s13287-020-02126-3 (PMC7805132; doi:10.1186/s13287-020-02126-3)
Supplement: Supplementary file 1 — Additional file 1. [file 13287_2020_2126_MOESM1_ESM.docx]

**Supplementary File**

**Methods**

***Phenotypic Screening Program*.** Human neural progenitor cells (hNPCs) (Lonza) were seeded (Biocoat, poly-L-Lysine coated, BectonDickenson) at 10000-20000 cells/well, grown in differentiation media (lacking bFGF) for four days, switched to neuron-maintenance media (Neurobasal™, Invitrogen) for up to 14 days *in vitro* (DIV). Cells were fed with 50% fresh media on DIV 1&3 with NNI-362 (1μM) and kinase inhibitors (>10nM). Then on DIV4, Biocoat plates were treated with *AlamarBlue* to determine percent increase in proliferation of NPCs. The plates were further incubated for up to 12DIV, then the cells were washed, fixed and treated with MAP2ab primary antibody (Abcam). Percent of new neurons was determined by a ratio of MAP2ab-stained cells over total nuclei (Hoechst 33342 dye, Abcam).

***Kinase profiling.*** NNI-362 (1µM) was tested against a panel of 151 kinases (Kinase Profile assay, CEREP). Reference standards were included in the assay and demonstrated the expected kinase activity modulation. Results showing an inhibition (or stimulation) ≤20% were considered not significant and attributable to signal variability.

***Phosphorylation p70S6 kinase****.* Cells were fixed at 5-6DIV or 11DIV, rinsed and stained with primary antibody to p70S6 kinase T421/S424 phosphorylation site (Cell Signaling Technology). The secondary antibody was an Alexa fluor-conjugated (Invitrogen). Using a Cellomic’s ArrayScan with Software, cells (nuclei stained-Hoechst dye) that co-stained with anti-p70S6 kinase determined the number of p70S6 kinase^+^ cells. For the relative peak activity at MAP kinases, cells were lysed at DIV5/6 or 11 and added to a membrane stamped with 26 antibodies (R&D Systems MAPK Proteome Profiler).

***Aging* in vivo *study.*** C57BL/6JNia male mice (20 or 3 months old) obtained from the National Institute on Aging were gavaged daily with vehicle (1% methylcellulose), or 10mg/kg NNI-351, or 1, 3, or 10 mg/kg NNI-362 for 4 weeks prior to and throughout behavioral testing.

***DS* in vivo *study***. VB6EiC3Sn.BLiA-Ts(17^16^)65Dn/DnJ and wild-type male mice (2 months old) obtained from Jackson Laboratories were injected ip with vehicle, NNI-362 (3mg/kg/day) or NNI-351 (3mg/kg/day).

***Immunohistology***. Subsets of mice received BrdU ip injections (50mg/kg) during the first 3 days of treatment. After perfusion, brains were sectioned (average of 5 coronal sections) and hippocampal subgranular and granular zones were stained (NeuroScience Associates, Knoxville, TN).

***Open Field test***. Each mouse explored a box with opaque walls and open top for 5min under dim illumination. Mobility was measured using Anymaze (Stoelting).

***Novel object recognition test.*** Acquisition: Mice explored a testing chamber with two identical objects placed equidistantly. Memory: Forty-eight hours after acquisition, mice explored the testing chamber with one familiar and one novel object (or similar object placed in novel position - Ts65Dn experiment). Mice explored for 7 minutes or until they had explored one or both objects for 30sec (nose within 1cm of the object). Percent time spent exploring the novel (object/location) vs. familiar object was calculated (Anymaze, Stoelting).

***Statistical Analyses***

*In vitro* studies, BrdU staining, and DS behavioral outcomes were analyzed using Kruskal-Wallis with Group as factor. Behavioral measurements for the aging study were subjected to one-way ANOVA with Group as factor. Alpha level was set at 0.05.
